# Global research trends on the associations between the microbiota and lung cancer: a visualization bibliometric analysis (2008–2023)

**DOI:** 10.3389/fmicb.2024.1416385

**Published:** 2024-08-30

**Authors:** Maoyuan Zhao, Jie Tian, Wang Hou, Liyuan Yin, Weimin Li

**Affiliations:** ^1^Lung Cancer Center, Frontiers Science Center for Disease-Related Molecular Network, West China Hospital, Sichuan University, Chengdu, Sichuan, China; ^2^Department of Thoracic Surgery, West China Hospital, Sichuan University, Chengdu, Sichuan, China; ^3^Lung Cancer Center, West China Hospital, Sichuan University, Chengdu, Sichuan, China; ^4^Department of Respiratory and Critical Care Medicine, Frontiers Science Center for Disease-Related Molecular Network, West China Hospital, Sichuan University, Chengdu, Sichuan, China; ^5^Precision Medicine Research Center, West China Hospital, Sichuan University, Chengdu, Sichuan, China; ^6^Research Units of West China, Chinese Academy of Medical Sciences, West China Hospital, Chengdu, Sichuan, China

**Keywords:** lung cancer, microbiota, bibliometric analysis, VOSviewers, Citespace

## Abstract

Numerous papers have been published on the microbiota in lung cancer in recent years. However, there is still a lack of bibliometric analysis of the microbiota in lung cancer in this field. Our paper did bibliometric analyses and elucidated the knowledge structure and study hotspots related to the microbiota in lung cancer patients. We screened publications reporting on the microbiota in lung cancer from 2008 to 2023 from the Web of Science Core Collection (WoSCC) database, and carried out bibliometric analyses by the application of the VOSviewers, CiteSpace and R package “bibliometrix.” The 684 documents enrolled in the analysis were obtained from 331 institutions in 67 regions by 4,661 authors and were recorded in 340 journals. Annual papers are growing rapidly, and the countries of China, the United States and Italy are contributing the most to this area of research. Zhejiang University is the main research organization. *Science* and *Cancer* had significant impacts on this area. Zhang Yan had the most articles, and the Bertrand Routy had the most co-cited times. Exploring the mechanism of action of the lung and/or gut microbiota in lung cancer and therapeutic strategies involving immune checkpoint inhibitors in lung cancer are the main topics. Moreover, “gut microbiota,” “immunotherapy,” and “short-chain fatty acids” are important keywords for upcoming study hotspots. In conclusion, microbiota research offers promising opportunities in lung cancer, with pivotal studies exploring the mechanisms that link lung and gut microbiota to therapeutic strategies, particularly through immune checkpoint inhibitors. Moreover, the gut-lung axis emerges as a novel target for innovative treatments. Further research is essential to unravel the detailed mechanisms of this connection.

## Introduction

According to recent estimates from 2023, lung cancer continues to account for approximately 1.8 million fatalities globally, underscoring its position as the leading cause of cancer-related deaths worldwide (World Health Organization, 2023). The intricate dance between genetic predispositions, environmental influences, and the microbiota’s role underscores the multifaceted nature of cancer etiology (Shieh et al., 2016; Goto, 2022). The human body’s microbial communities, integral to the host’s microenvironmental stability, serve as double-edged swords in health and disease. While essential for maintaining physiological homeostasis, their imbalance has been implicated in a spectrum of pathologies, including oncogenesis (Garrett, 2015; Zitvogel et al., 2018). The revelation that microbial dysbiosis can influence cancer development through various mechanisms—ranging from inflammation modulation and carcinogenic metabolite production to genotoxicity and cell cycle disruption—highlights the microbiome’s critical role in cancer (Mao et al., 2018). Recent advancements in next-generation sequencing (NGS) have debunked the once-held belief that lung tissues are sterile, uncovering a complex microbiome within the lungs (Segal et al., 2013).

Comparative analyses of the lung and oropharyngeal microbiotas in both healthy controls and lung cancer patients reveal a predominance of similar bacterial phyla, such as *Firmicutes*, *Proteobacteria*, and *Bacteroidetes* (Morris et al., 2013; Dickson et al., 2015; Bassis et al., 2015). However, the microbial ecosystems in lung cancer patients exhibit dysbiosis, with altered abundances of these phyla, particularly an increase in *Proteobacteria*, which may contribute to the tumor microenvironment and cancer progression (Morris et al., 2013; Bassis et al., 2015; Marsh et al., 2016). These findings suggest the presence of low-density, diverse microbial ecosystems within lung tissue, bronchoalveolar lavage (BAL) fluid, and sputum, maintained through a delicate balance of microbial migration and clearance (Morris et al., 2013; Bassis et al., 2015; Marsh et al., 2016). Furthermore, the lung microbiota has been linked to the risk of chronic obstructive pulmonary disease (COPD), with specific genera such as *Prevotella*, *Streptococcus*, and *Haemophilus* showing significant variations between COPD patients and healthy controls, especially during COPD exacerbations (Kim et al., 2017; Sze et al., 2012; Garcia-Nuñez et al., 2014). Moreover, the association between certain microorganisms and an increased risk of lung cancer (Garrett, 2015), alongside the correlation between antibiotic use and lung cancer susceptibility, underscores the impact of microbial imbalances on cancer risk (Kim et al., 2023). Additionally, the inflammation induced by microorganisms, via mechanisms like toll-like receptor activation, underscores a pathway through which the microbiota can influence lung carcinoma development (Chen et al., 2022; Orlacchio and Mazzone, 2021).

Understanding the global research landscape on the microbiota’s role in lung cancer is crucial, given its significant implications for identifying potential therapeutic targets and biomarkers. In our study, we utilized CiteSpace and VOSviewer as the primary tools for conducting bibliometric analysis and visualizing the literature landscape related to lung cancer and the microbiota. These software tools were instrumental in applying both quantitative and qualitative methodologies to map out key research trends, collaboration networks, and emerging topics within the field. Previous studies, including the work by Sun et al. (Alam et al., 2024), have explored broader relationships involving the gut microbiota and lung diseases. In contrast, our study specifically examines research trends and collaborations focused on the microbiota associated with lung cancer. By narrowing the focus to this specific area, our analysis reveals unique patterns and emerging trends that contribute to a deeper understanding of the microbiota’s role in lung cancer development and progression.

This study not only aims to consolidate the existing body of knowledge but also to identify research hotspots and gaps that can guide future investigations. By doing so, it provides valuable insights that could inform subsequent research efforts and support the development of microbiota-targeted therapies or diagnostics, ultimately enhancing the management and treatment of lung cancer.

## Materials and methods

### Publication search

**Figure 1:**
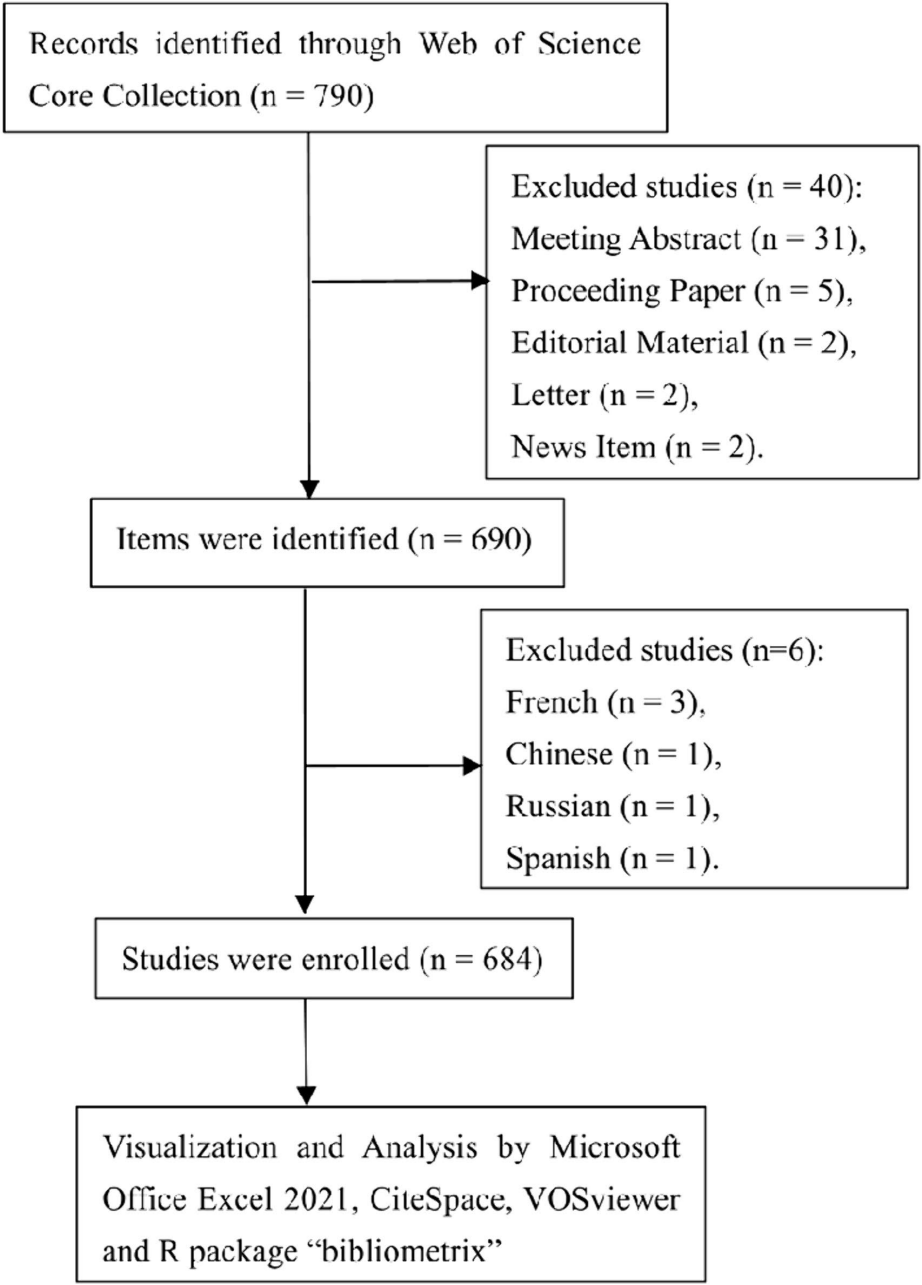
Publications screening flowchart.

Our search strategy commenced with an exhaustive review of literature indexed in the Web of Science Core Collection (WoSCC) database. Utilizing the search query “{lung cancer} AND {microbiota},” we identified publications dated from January 1, 2003, to October 31, 2023. An initial tally yielded 730 studies. We refined our pool by selecting only “Article” and “Review Article” types, which resulted in 690 publications. Further screening for English-language papers led to the exclusion of 6 studies, culminating in a final count of 684 eligible studies for inclusion (Figure 1).

### Data analysis

To dissect the amassed literature, we employed VOSviewer (version 1.6.19) and CiteSpace (version 6.2 R4), alongside the R package “bibliometrix” (version 4.3.1), for an in-depth bibliometric analysis. VOSviewer facilitated the construction of bibliometric networks, offering visual representations of the interplay between countries, institutions, journals, and authors, based on data such as citations, co-citations, bibliographic couplings, and co-authorship ties. This software visualizes the networks through nodes representing different entities (e.g., keywords, regions, institutions) and lines indicating the strength of relationships (e.g., collaboration or co-citation). The node size and color denote the frequency and category of the entities, respectively. CiteSpace was utilized to generate a dual-map overlay of sources and references, highlighting citation bursts and evolving trends within the field. This analysis provided a macroscopic view of the literature’s foundation and emerging focal points (Synnestvedt et al., 2005; Pan et al., 2018). Further, the “bibliometrix” R package enabled the examination of trending topics and the creation of global distribution maps, offering insights into the geographical spread of research activity (Aria and Cuccurullo, 2017). Complementary to these tools, Microsoft Office Excel 2021 supported our quantitative analysis, facilitating the organization and presentation of publication data.

## Results

### Overview of publications

**Figure 2:**
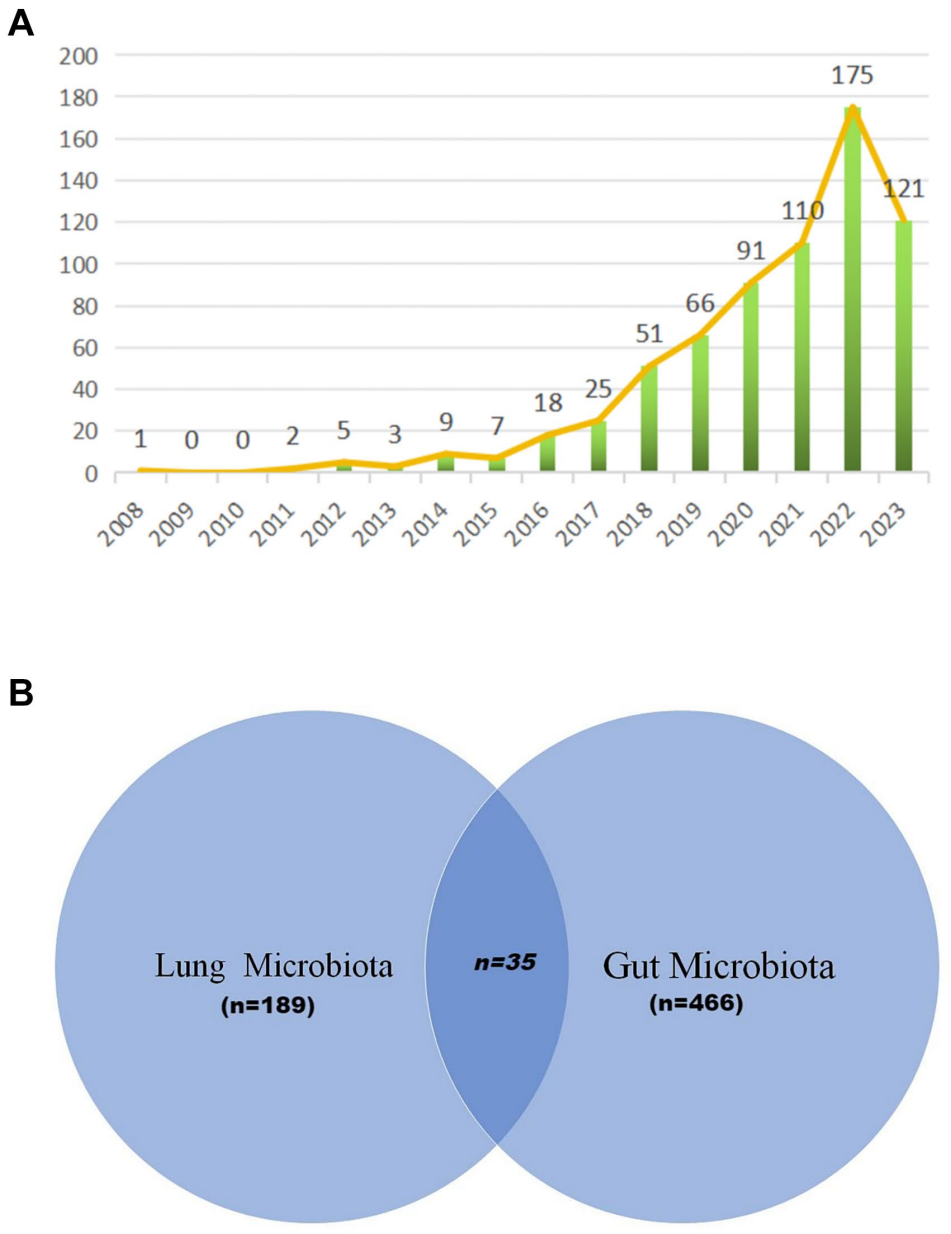
Annual output of research of microbiota in lung cancer. **(A)** The visualization by Venn diagram provides a comprehensive overview of the existing research landscape, illustrating the areas of focus within the microbiota-lung cancer field **(B)**.

Our bibliometric analysis incorporated 684 documents, originating from 331 institutions across 67 countries, authored by 4,661 researchers, and published in 340 journals. The timeline for these publications spanned from 2008 to 2023, with the inaugural paper emerging in 2008. Notably, a hiatus in publications was observed in the subsequent 2 years. The early phase (2011–2015) witnessed modest activity, with annual outputs ranging from 2 to 9 papers. A significant uptick in publications began in 2016, indicating a burgeoning interest in this field. The period from 2016 to 2022 was characterized by an average annual growth rate of 45.39% and an average output of 76.57 papers. This momentum continued, with 2023 seeing 121 publications as of October 31, 2023 (Figure 2A). Moreover, we observed distinct focuses on the lung microbiota, the gut microbiota, and studies that consider both. As depicted in Figure 2B, 189 studies were dedicated exclusively to the lung microbiota, 466 studies focused on the gut microbiota, and 35 studies explored the relationship between both. This distribution underscores the significant research emphasis on the lung/gut microbiota, while also highlighting the interdisciplinary studies that investigate both microbiota.

### Geographic and institutional contributions

**Figure 3:**
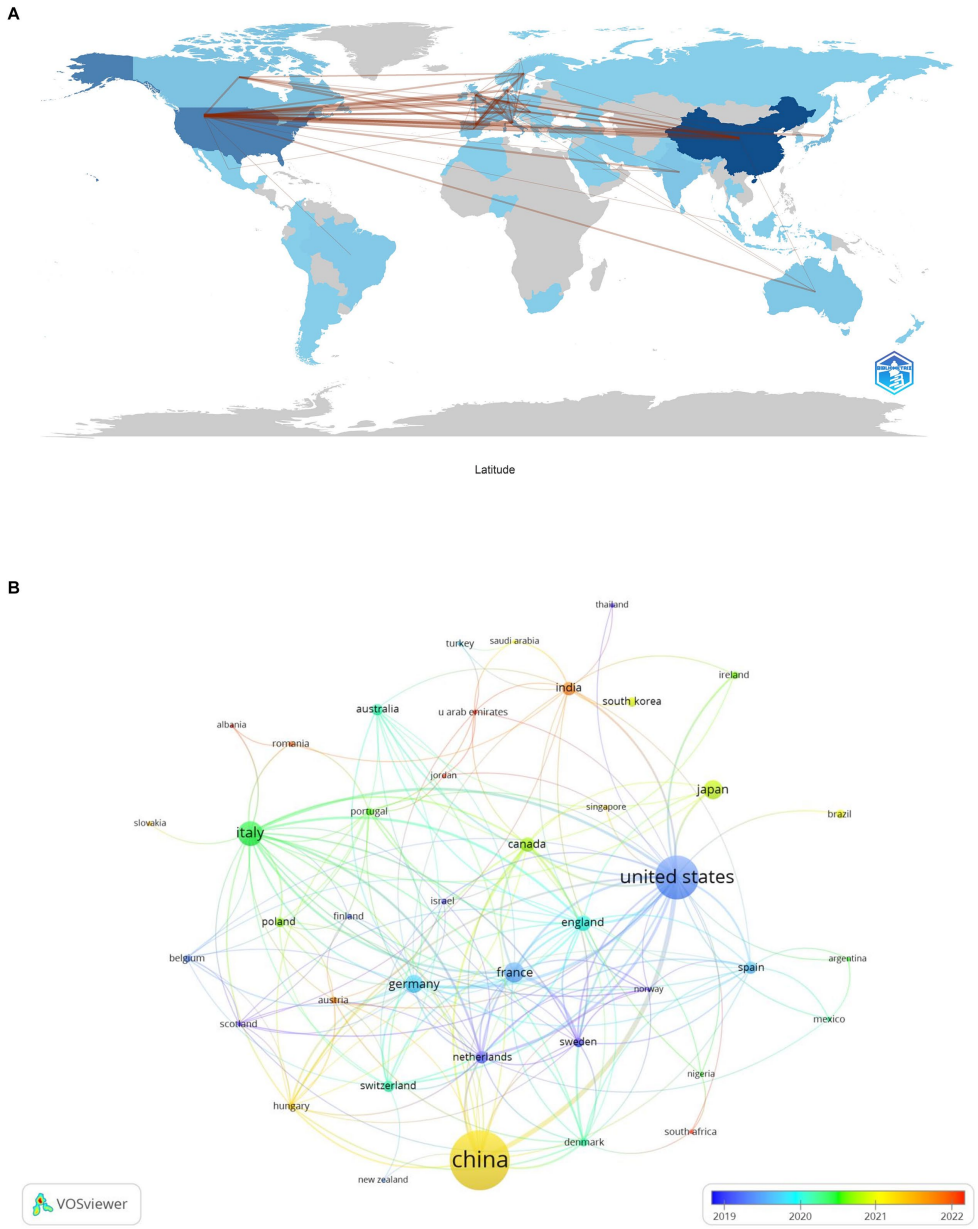
The geographical distribution **(A)** and visualization of countries **(B)** on research of microbiota in lung cancer.

Our analysis on lung cancer and microbiota literature spans across 331 institutions from 67 countries. We mapped the global research distribution, revealing significant contributions from regions across Europe, Asia, and North America (Figure 3). The forefront of this research is led by China (*n* = 279), the United States (*n* = 163), Italy (*n* = 60), France (*n* = 40), and Japan (*n* = 38). Notably, publications from North American and European entities exhibit a higher citation rate per publication compared to those from Asian counterparts. It is important to note that while citation rates serve as an indicator of the influence and impact of research, they are an indirect measure and do not directly assess the biological associations between the microbiota and lung cancer. Higher citation rates may suggest that the work has contributed significantly to advancing our understanding of these associations, but they do not alone establish a direct link. Further biological and clinical studies are necessary to explore these connections in depth.

**Figure 4:**
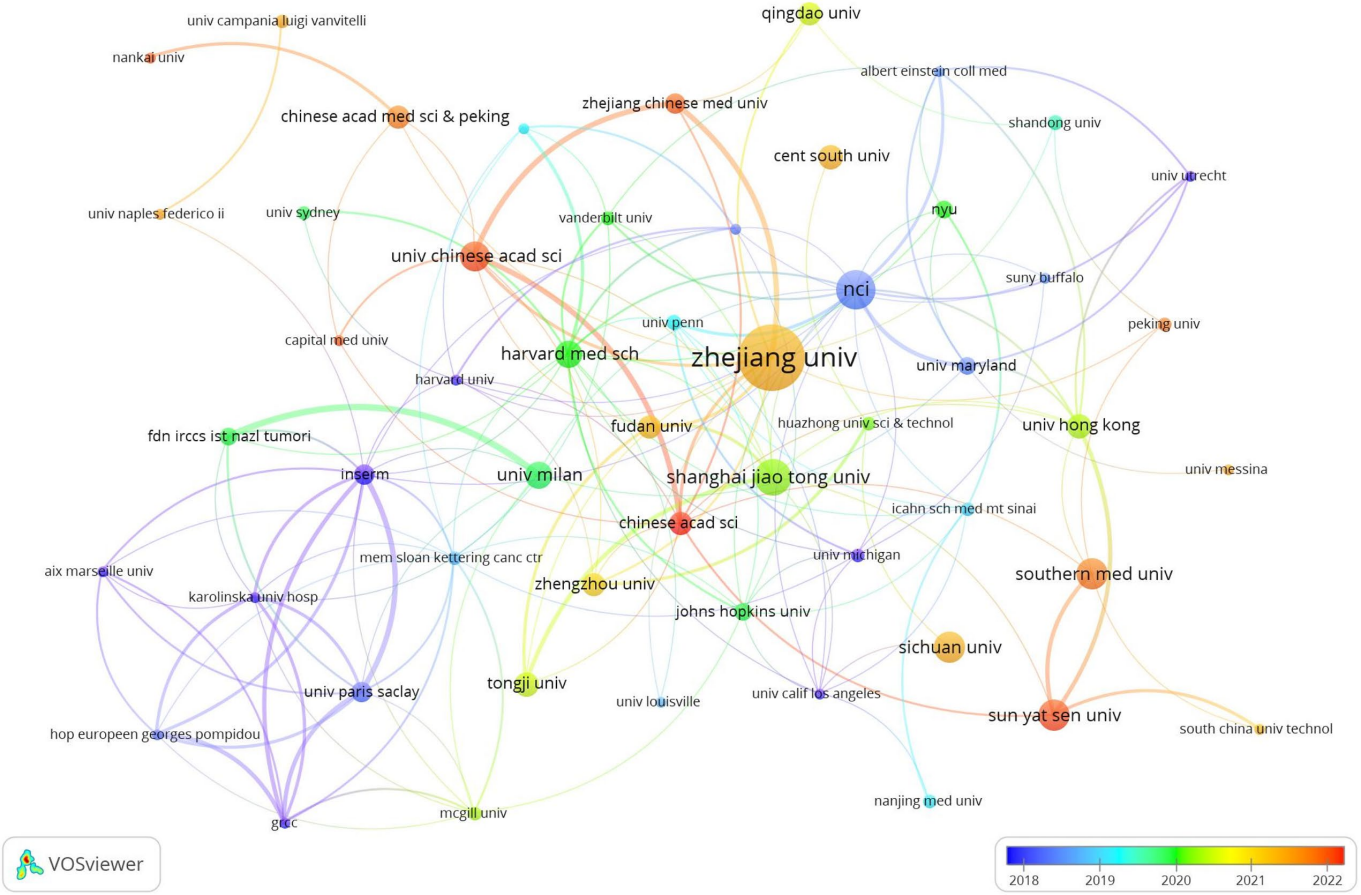
The visualization of institutions on research of microbiota in lung cancer. This study selected 53 institutions based on the minimum number of publications equal to 5 for visualization, and constructed a collaborative network based on the number and relationship of publications of each institution.

Table 1 highlights that the majority (70%) of the leading institutions are based in China, the United States, Ireland, and Italy. Among these, Zhejiang University (*n* = 29), the National College of Ireland (*n* = 17), and Shanghai Jiao Tong University (*n* = 16) emerged as the top contributors. A collaboration network analysis among 53 select institutions showed a particularly strong partnership among Zhejiang University, the University of Chinese Academy of Science, and Zhejiang Chinese Medical University. Moreover, six organizations, including the University of Chinese Academy of Sciences, National College of Ireland, Harvard Medical School, Zhejiang University, Inserm, and Shanghai Jiao Tong University, demonstrated a significant impact through both prolific publication records and extensive collaborations (Figure 4).

**Table 1:** Top 10 countries and organizations on the research of microbiota in lung cancer.

Rank	Country	Counts	Citations	Average citation/publications	Organization	Counts	Citations
1	China (Asia)	279	6,476	23.21	Zhejiang University (China)	29	669
2	The United States (North America)	163	14,892	91.36	National College of Ireland (Ireland)	17	1,499
3	Italy (Europe)	60	2,608	43.47	Shanghai Jiao Tong University (China)	16	944
4	France (Europe)	40	5,152	128.8	Sichuan University (China)	14	267
5	Japan (Asia)	38	879	22.08	Southern Med university (China)	14	222
6	Germany (Europe)	34	1,412	41.53	Sun Yat-sen University (China)	14	498
7	England (Europe)	25	1,022	40.88	University of Chinese Academy of Sciences (China)	13	77
8	Canada (North America)	23	2,014	87.56	Harvard Medical School (The United States)	12	247
9	India (Asia)	19	134	7.05	The University of Milan (Italy)	12	539
10	Spain (Europe)	18	568	31.55	Central South University (China)	11	141

### Journal contributions and co-citations

**Figure 5:**
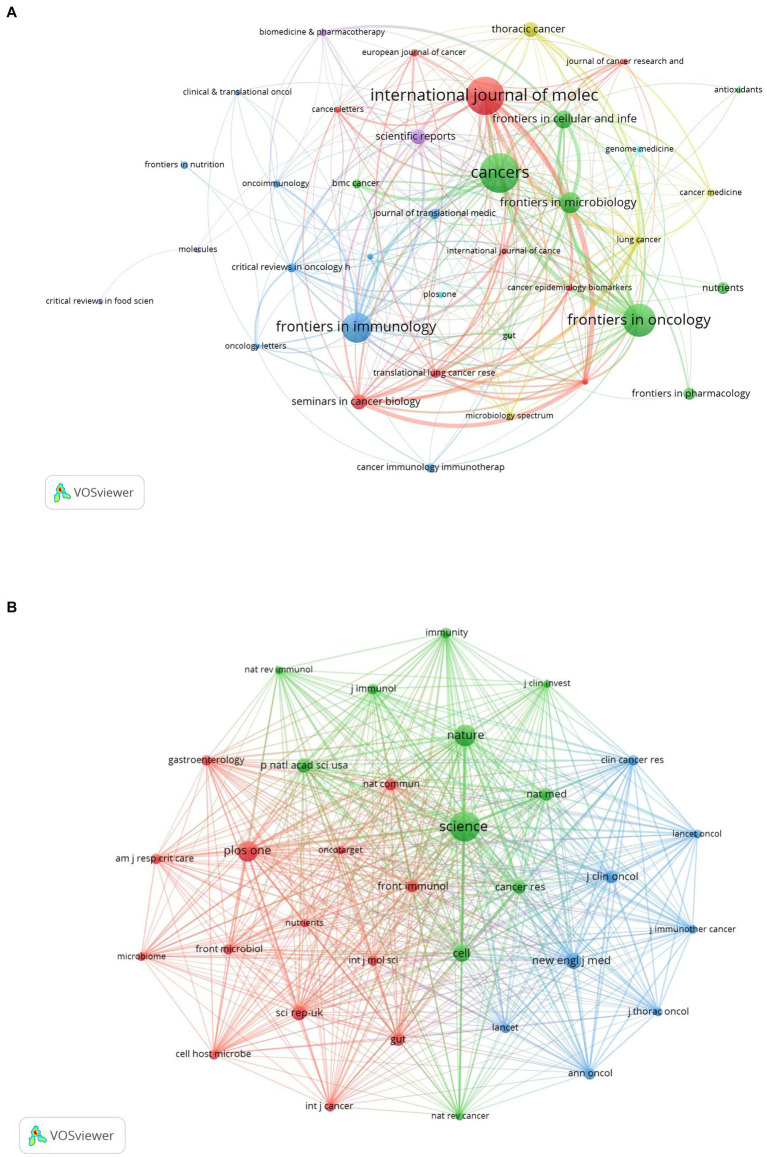
The visualization of journals **(A)** and co-cited journals **(B)** on research of microbiota in lung cancer. **(A)** This study enrolled 37 journals based on the minimum number of relevant publications equal to four and mapped the journal network. **(B)** More than 300 co-citation journals (33 journals) were filtered to map the co-citation network.

The discourse on microbiota in lung cancer encompasses 340 journals, with the top 15 outlined in Table 2. *Cancers* leads with the highest contribution (*n* = 27), followed by the *International Journal of Molecular Science* (*n* = 26), *Frontiers in Oncology* (*n* = 22), *Frontiers in Immunology* (*n* = 20), and *Frontiers in Microbiology* (*n* = 14). Among these, *Seminars in Cancer Biology* boasts the highest impact factor (IF = 14.5), with the *Journal of Translational Medicine* following (IF = 7.4). A collaborative mapping of 37 selected journals revealed strong citation connections, particularly *Cancers* with the *International Journal of Molecular Sciences*, *Frontiers in Oncology*, *Frontiers in Immunology other* (Figure 5A).

**Table 2:** Top 15 journals and co-cited journals on the research of microbiota in lung cancer.

Rank	Journal	Counts	Citations	IF^a^	Q^b^	Co-cited journal	Co-citation	IF^a^	Q^b^
1	Cancers	27	265	5.2	2	Science	2,272	56.9	1
2	International Journal of Molecular Sciences	26	373	5.6	1	Nature	1,321	64.8	1
3	Frontiers in Oncology	22	215	4.7	2	PLoS One	1,256	3.7	2
4	Frontiers in Immunology	20	288	7.3	1	Cell	910	64.5	1
5	Frontiers in Microbiology	14	552	5.2	2	The New England Journal of Medicine	875	158.5	1
6	Frontiers in Cellular and Infection Microbiology	12	223	5.7	1	Proceedings of the National Academy of Sciences of the United States of America (Proc Natl Acad Sci U S A)	751	11.1	1
7	Scientific Reports	10	471	4.6	2	Scientific Reports	714	4.6	2
8	Seminars in Cancer Biology	10	309	14.5	1	Nature Medicine	606	82.9	1
9	Thoracic Cancer	10	91	2.9	3	Cancer Research	603	11.2	1
10	Frontiers in Pharmacology	8	145	5.6	1	Journal of Clinical Oncology	598	45.3	1
11	Nutrients	8	173	5.9	1	Gut	582	24.5	1
12	Journal of Translational Medicine	7	256	7.4	1	Frontiers in Immunology	559	7.3	1
13	BMC Cancer	6	53	3.8	2	Nature Communications	536	16.6	1
14	Cancer Immunology Immunotherapy	6	116	5.8	1	Annals of Oncology	501	50.5	1
15	Critical Reviews in Oncology Hematology	6	203	6.2	1	Immunity	461	32.4	1

a The impact factor of the journal are obtained from Journal Citation Reports 2023.

b The quartile of the journal are obtained from Journal Citation Reports 2023.

**Figure 6:**
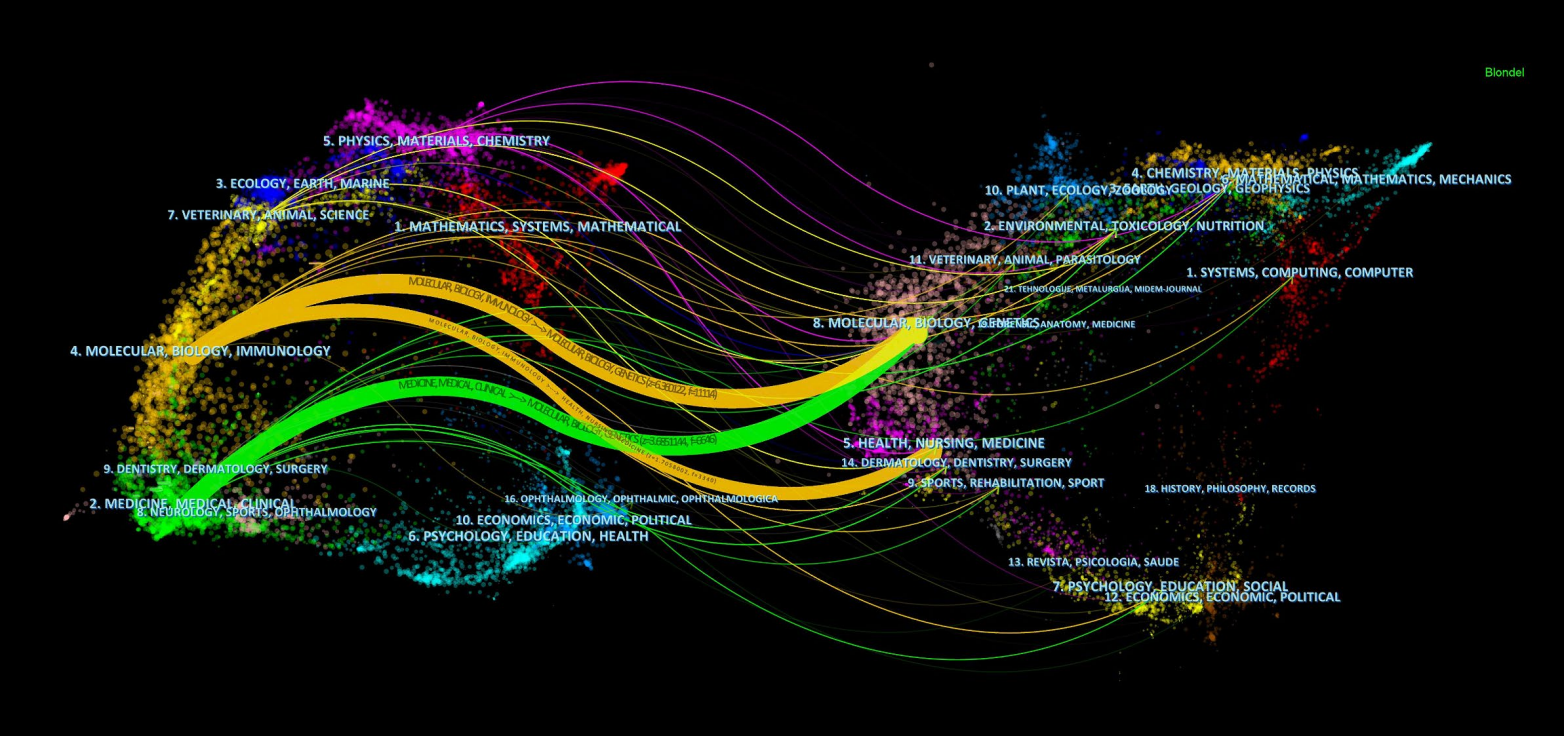
The dual-map overlay of journals on research of microbiota in lung cancer, with clusters of citing journals on the left and clusters of cited journals on the right.

In terms of co-citations, 80% of the top 15 journals were cited over 500 times, with *Science* (co-citation = 2,272) leading, followed by *Nature* (co-citation = 1,321) and *PLoS One* (co-citation = 1,256). *The New England Journal of Medicine* stands out with the highest impact factor (158.5), closely followed by *Nature Medicine* (IF = 82.9), *Nature* (IF = 64.8), and *Cell* (IF = 64.5). A co-citation network from 33 journals underscored the interconnectedness of the field, especially between *Science* and journals like *Nature*, *Cell*, *Nature Medicine*, and *Cancer Research* (Figure 5B). The dual-map overlay illustrates the predominant citation trajectory from publications in Molecular/Biology/Genetics and Health/Nursing/Medicine to those in the Molecular/Biology/Immunology sector (Figure 6), highlighting a significant cross-disciplinary engagement (Wu et al., 2022)

### Leading contributors and collaborative efforts

**Figure 7:**
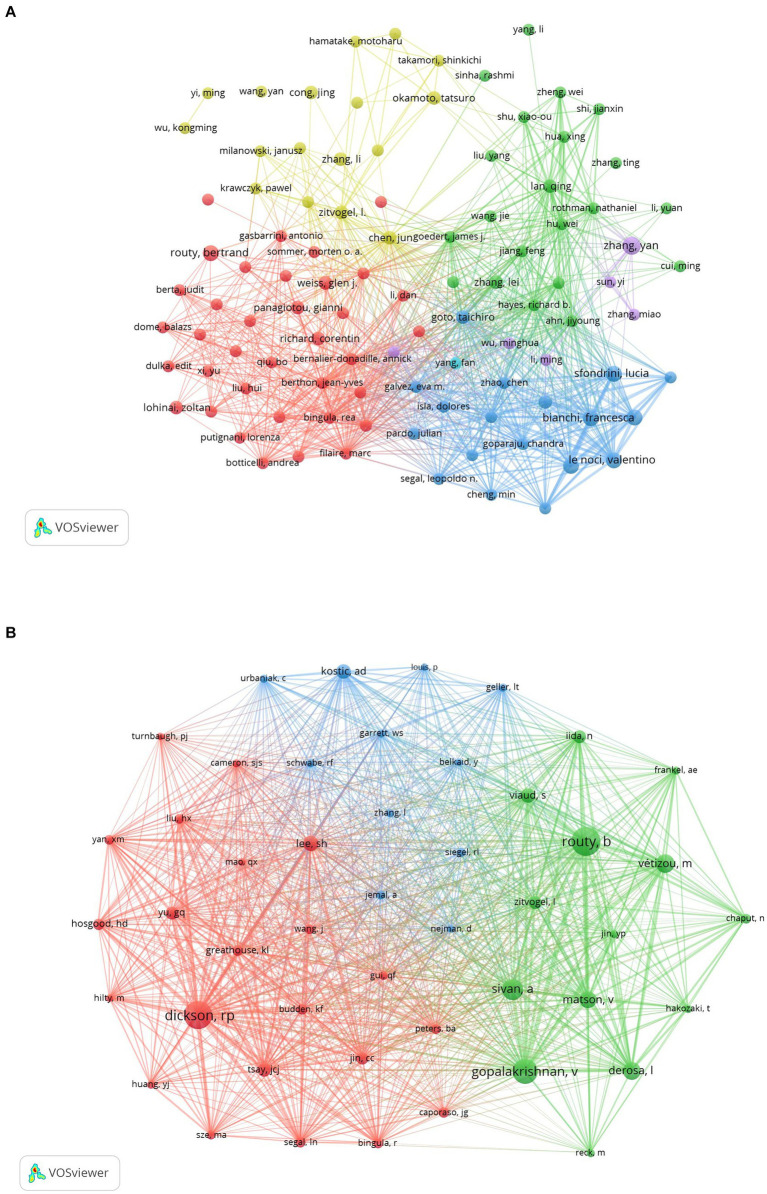
The visualization of authors **(A)** and co-cited authors **(B)** on research of microbiota in lung cancer. **(A)** A collaborative network was constructed based on researchers whose number of published documents is more than or equal to three. **(B)** This study selected 47 authors to map the co-citation network based on minimum co-citations equals to 50.

The collective insights into the microbiota’s role in lung cancer have been shaped by the contributions of 4,661 authors. Among these, the top 10 authors each have authored at least five publications (Table 3). A collaborative map, based on the volume of research output, illustrates the network of these prolific authors (Figure 7A). Zhang Yan emerges as a leading figure with the highest number of publications, followed closely by notable researchers such as Bianchi Francesca, Le Noci Valentino, Routy Bertrand, Sfondrini Lucia, Sommariva Michele, and Tagliabue Elda. This network also highlights extensive collaborations, particularly among researchers like Sommariva Michele, Bianchi Francesca, Sfondrini Lucia, and Guglielmetti Simone.

**Table 3:** Top 10 authors and co-cited authors on research of microbiota in lung cancer.

Rank	Author	Counts	Co-cited Authors	Citations
1	Zhang, Y	6	Routy, B	238
2	Bianchi, F	5	Dickson, RP	228
3	Valentino, LE	5	Gopalakrishnan, V	201
4	Bertrand, R	5	Sivan, A	167
5	Lucia, S	5	Vétizou, M	140
6	Michele, S	5	Derosa, I	137
7	Elda, T	5	Matson, V	131
8	Chen, J	4	Lee, Sh	116
9	Cong, J	4	Viaud, S	110
10	Taichiro, G	4	Kostic, Ad	107

As shown in Table 3, we found that all 10 authors were co-cited at least 100 times; of these, Routy B (*n* = 238) was the most co-cited author, followed closely by Dickson RP (*n* = 228) and Gopalakrishnan V (*n* = 201). Furthermore, we constructed a co-citation network selected from 47 authors on the basis of parameter settings. As shown in Figure 7B, we also found numerous close collaborations among multiple co-cited researchers, for instance, Routy B and Gopalakrishnan V, Dickson RP and Huang YJ.

### Top co-cited references

**Figure 8:**
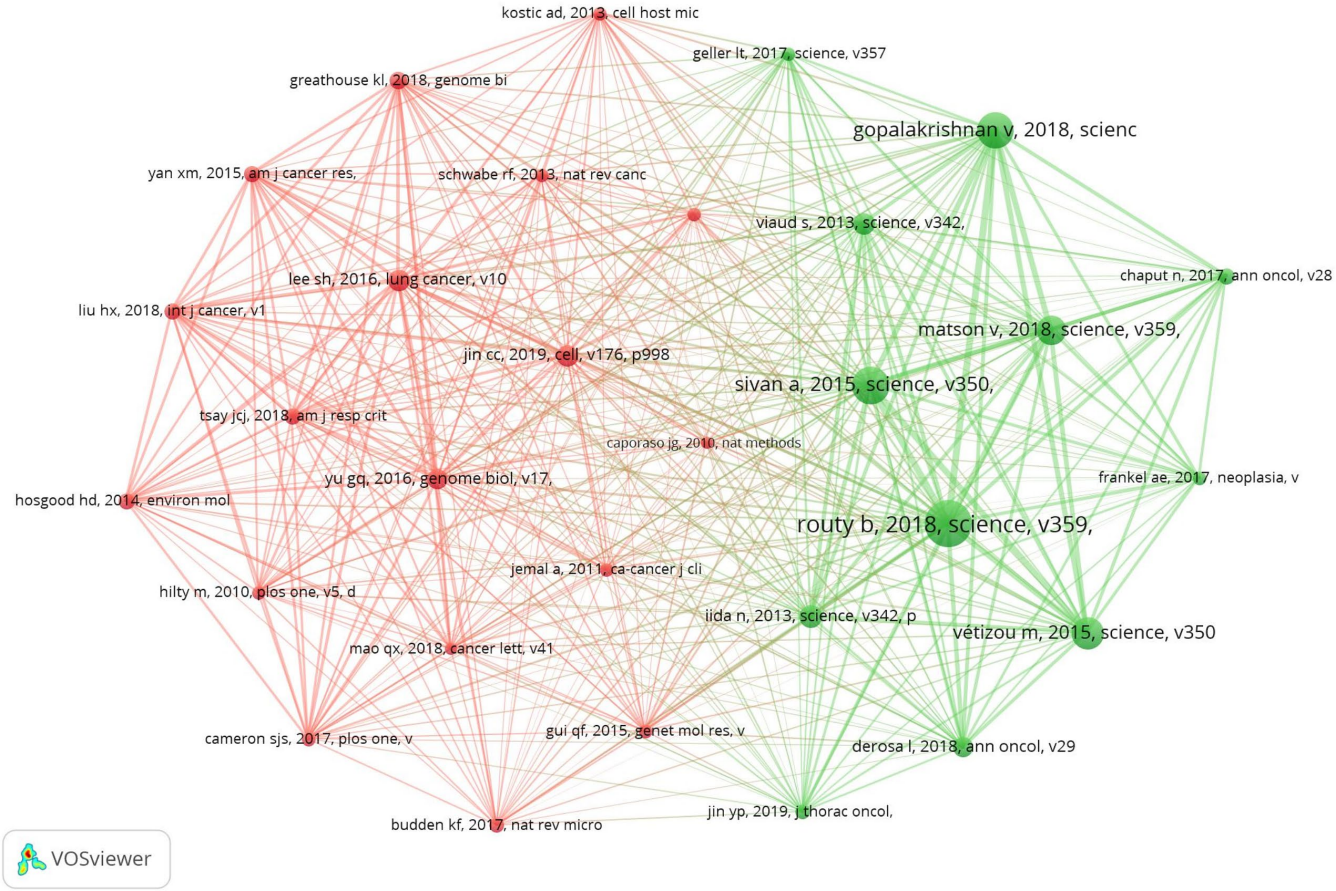
The visualization of co-cited references on research of microbiota in lung cancer.

Our research database encompasses 39,752 co-cited references, with the top 10 most influential references identified as being cited more than 100 times each (Supplementary Table 1). A co-citation map of 30 significant references—each cited at least 45 times—demonstrates the intricate connections within this field (Figure 8). In addition, “Routy H, 2018, Science” has closely co-cited collaborations with “Gopalakrishnan V, 2018, science,” “Sivan A, 2015, science,” “Matson V, 2018, science,” “Vétizou M, 2015, science.”

### References with citation bursts

In the study, we utilized CiteSpace software to conduct a citation burst analysis, which reveals references experiencing a sharp increase in citations within a specified period, signifying their significant impact. This analysis yielded a network of references characterized by pronounced citation bursts, from which we discerned the 20 most prevalent instances, marked by red bars. These bursts spanned from 2014 to 2018, indicating a concentrated period of heightened interest. Among these, two references stood out with burst strengths exceeding 20. The foremost reference, “Commensal Bifidobacterium promotes antitumor immunity and facilitates anti-PD-L1 efficacy” by Ayelet Sivan et al., demonstrated a burst strength of 24.37 during 2016–2020. Following closely was “Anticancer immunotherapy by CTLA-4 blockade relies on the gut microbiota” by Marie Vétizou et al., published in *Science*, with a strength of 21.66 and a similar burst period. The burst strength across the top 20 references varied from 4.75 to 24.37, with durations extending from 2 to 5 years. We also encapsulated the core content of these significant references in Supplementary Table 2.

**Figure 9:**
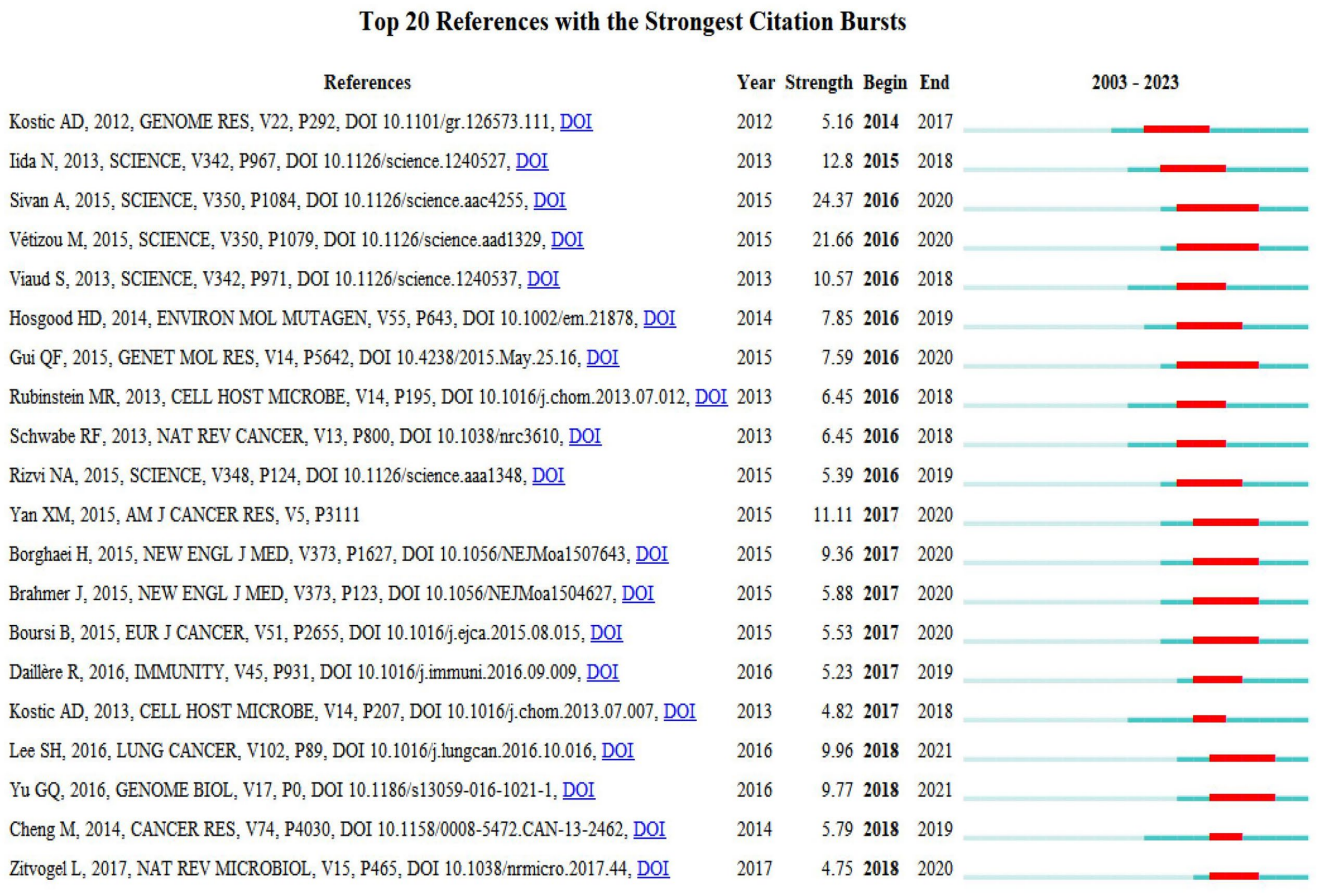
Top 20 references with strong citation bursts. A red bar indicates high citations in that year.

The analysis of global research contributions, including the geographic and institutional distribution, provides a foundational understanding of the landscape in which microbiota and lung cancer research is being conducted. This study highlights the key players and regions contributing to the field, which is essential for contextualizing the emerging trends in research that directly explore the associations between the microbiota and lung cancer. In the following sections, we delve into these research trends, focusing on how the microbiota is increasingly recognized as a critical factor in lung cancer development and progression (Figure 9).

### Analysis of hotspots and frontiers

**Figure 10:**
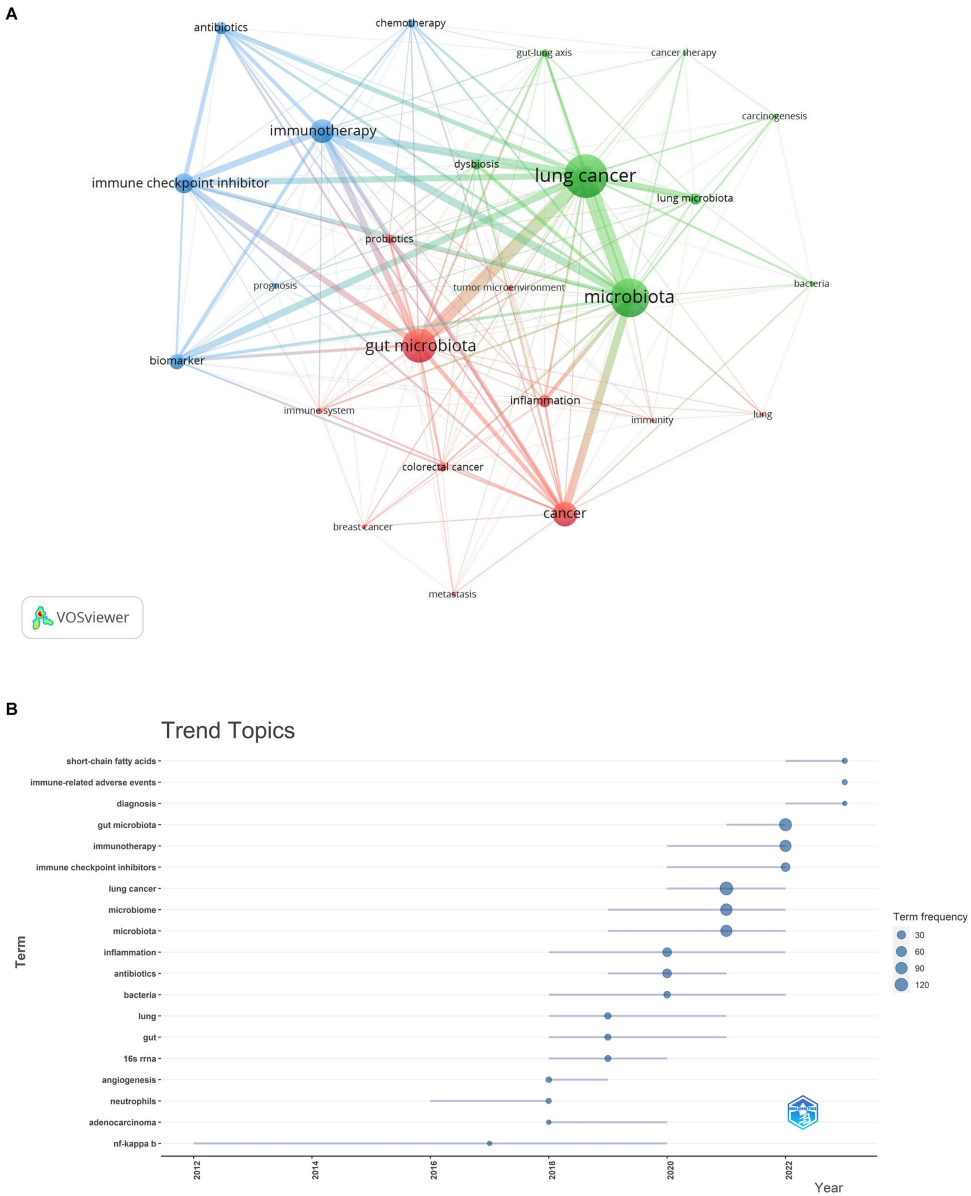
Keyword cluster analysis **(A)** and trend topic analysis **(B)**.

Our analysis extended to identifying research hotspots and emerging frontiers through keyword co-occurrence analysis. This methodology enabled us to pinpoint the predominant themes and cutting-edge topics within specific domains, as illustrated in Figure 10A. Among the top 20 recurrent keywords—some appearing over 60 times—were pivotal terms like gut microbiota, cancer, immunotherapy, and immune checkpoint inhibitor (ICI) (Table 4). These terms underscore the principal research direction focusing on the microbiota’s role in lung cancer. The network was categorized into three clusters (Figure 10A), representing distinct research domains: green clusters encompassed terms related to lung cancer, microbiota, the gut-lung axis, and cancer therapy; red clusters focused on the gut microbiota, the immune system, and the tumor microenvironment; and blue clusters were dedicated to immunotherapy, biomarkers, and related themes.

**Table 4:** Top 20 keywords on research of microbiota in lung cancer.

Rank	Keywords	Counts	Rank	Keywords	Counts
1	Lung cancer	185	11	Dysbiosis	27
2	Microbiota	158	12	Colorectal cancer	23
3	Gut microbiota	131	13	Probiotics	23
4	Cancer	86	14	Chemotherapy	21
5	Immunotherapy	82	15	Gut-lung axis	19
6	Immune checkpoint inhibitor	66	16	Immune system	12
7	Biomarker	46	17	Prognosis	12
8	Inflammation	36	18	Tumor microenvironment	12
9	Antibiotics	34	19	Bacteria	11
10	Lung microbiota	31	20	Carcinogenesis	11

As illustrated in Figure 10B, the keyword analysis reveals that from 2012 to 2016, discussions predominantly centered around nuclear factor-kappa B. Following this, from 2016 to 2018, the focus shifted toward neutrophil information. During this period, scholars expanded their research horizons, delving into various directions such as inflammation, bacteria, lung and gut health, 16S rRNA, angiogenesis, and adenocarcinoma. By 2019, the academic community further broadened its exploration, examining the intricate relationships between the microbiota, lung cancer, and bacteria. In subsequent years, attention turned toward the diagnosis of microbiota in lung carcinoma and the compilation of its therapeutic potential in treating lung carcinoma. Additionally, keywords related to gut microbiota, immunotherapy, and short-chain fatty acids (SCFAs) garnered significant interest during 2022–2023, indicating their emergence as potential research hotspots in the context of microbiota and lung cancer.

## Discussion

### General information

This study provides a focused bibliometric analysis specifically on the microbiota in lung cancer, covering publications from 2008 to 2023. Initially, research in this area was sparse, spanning from 2008 to 2015. However, a significant increase in publication volume occurred after 2016, with an average annual growth rate of 45.39% and an average of 76.57 publications per year until 2022. The surge in publications in 2022 highlights the growing recognition of microbiota as a promising research area in lung cancer. The primary contributors to this field include China, the United States, Ireland, and Italy, with the most prolific institutions being Zhejiang University, the National College of Ireland, and Shanghai Jiao Tong University. Notably, collaboration among Zhejiang University, the University of Chinese Academy of Science, and Zhejiang Chinese Medical University has been particularly robust.

The prominence of journals such as *Science* and *Cancers* in publishing research on the microbiota in lung cancer underscores the growing acknowledgment of the microbiome’s role in oncology. The high citation rate of these journals highlights their impact on shaping current research directions and methodologies. Zhang Yan’s contributions, particularly in elucidating the characteristics of lung microbiota in advanced-stage lung cancer, provide valuable insights into potential biomarkers for prognosis and therapeutic targets. The correlation between the microbiota in tumor tissues and BALF suggests a systemic influence of the microbiome on lung cancer pathophysiology. This finding could lead to novel diagnostic tools that use microbial signatures to enhance the accuracy of lung cancer staging and potentially guide personalized treatment strategies (Zhang et al., 2022a,b). Furthermore, the work of Marie-Vétizou and colleagues has significantly advanced our understanding of the microbiome’s influence on cancer therapy. Their studies on the effects of CTLA-4 blockade on *Bacteroidales* and the subsequent immunostimulatory responses (Marie-Vétizou et al., 2015) indicate that manipulating the microbiome could enhance the efficacy of immune checkpoint inhibitors. The potential for microbiome modulation to affect cancer prognosis by altering immunosuppression and the tumor microenvironment suggests new avenues for integrative therapies that combine microbiome regulation with conventional and immunotherapeutic approaches (Pitt et al., 2016; Zitvogel et al., 2017; Fidelle et al., 2023). This research also implicates the microbiome in the modulation of cancer-related pathways beyond the immune response, including tumor cell signaling and metabolic processes. Marie-Vétizou’s exploration of microbial community checkpoints and their role in directing T cells to various cancer types presents a promising frontier for targeted immunotherapy, offering a potentially transformative impact on treatment paradigms for lung and other cancers. In conclusion, the intersections of microbiota research with lung cancer pathogenesis and treatment point to a rich field of study that may yield critical advances in oncological therapies. Future research should aim to clarify the mechanisms through which the microbiome influences tumor behavior and resistance to therapies, paving the way for microbiome-based diagnostics and therapeutics.

### Research basis

Co-cited references provide a foundational basis for understanding a research field (Chen, 2006). The synthesis of findings from these co-cited references underscores a significant trend in microbiota research related to lung cancer: the exploration of microbial influence on immune modulation and cancer progression. Fidelle et al.’s identification of microbial checkpoints provides a molecular basis for how the intestinal microbiota might influence systemic immune responses, particularly in the context of cancer immunosuppression. This work is instrumental in paving the way for novel therapeutic strategies that harness the microbiome to boost the effectiveness of existing cancer treatments (Fidelle et al., 2023). The work by Sivan et al. adds another layer of complexity, revealing how certain commensal bacteria can naturally enhance the body’s ability to fight cancer through immune pathways. Their findings suggest that augmenting these natural allies within our microbiota could be a viable method to increase the efficacy of checkpoint inhibitors like anti-programmed cell death protein 1 ligand 1 (PD-L1), a popular cancer therapy (Sivan et al., 2015). Lee et al.’s research provides practical insights into the diagnostic potential of microbiota analysis. By identifying specific bacterial species as biomarkers, this approach could lead to more precise and early detection of lung cancer, distinguishing malignant growths from benign lesions based solely on microbial composition (Lee et al., 2016). Lastly, Jin et al.’s study highlights the direct inflammatory roles played by local microbiota in promoting lung carcinoma. This connection between local microbiota and inflammation introduces potential targets for interventions that could mitigate inflammation and, consequently, reduce cancer progression (Jin et al., 2019). Collectively, these co-cited references highlight four central themes: commensal microbiota, intestinal microbiota, bacterial community components, and antitumor/cancer immunotherapy strategies. Furthermore, these findings collectively point to a transformative potential in cancer therapy and diagnosis, influenced by the intricate interactions between microbiota and the host immune system. Future research should focus on the mechanistic pathways through which microbiota influence cancer and the potential for clinical applications that modify the microbiome to fight cancer more effectively. This could include the development of microbial-based therapies, enhanced diagnostic tools, and integrated treatment protocols that consider the microbiome as a critical element in patient management and therapy design.

### Hotspots and frontiers

Researchers often use references with citation bursts and keyword co-occurrence analysis to quickly identify hotspots and frontiers in specific fields. Currently, major topics in lung cancer research related to microbiota focus on the lung and gut microbiota and anticancer immunotherapy.

#### Lung microbiota

Recent studies have investigated the relationship between lung cancer stage and the composition of the microbiota in lung tissues. These studies suggest that the microbiota may vary depending on the stage of lung cancer. For instance, certain microbial taxa, such as Proteobacteria, have been found in higher relative abundance in more advanced stages of lung cancer. Additionally, differences in microbial diversity have been observed, with lower alpha diversity often associated with more advanced stages. These findings indicate that there may be a significant relationship between lung cancer progression and changes in the lung microbiota (Karvela et al., 2023; Whiteside et al., 2021). Furthermore, studies have identified that the composition of the lung microbiota can indeed vary between different types of lung cancer, such as non-small cell lung cancer (NSCLC) and small cell lung cancer (SCLC). For example, Streptococcus and Veillonella have been associated with NSCLC, particularly in advanced stages. On the other hand, the lower airway microbiota composition has been observed to differ between NSCLC and SCLC, suggesting that each type of lung cancer might have distinct microbiota profiles that could influence disease progression and response to treatment (Najafi et al., 2021; Chaturvedi et al., 2010). Contrary to earlier beliefs that lung tissues are sterile, recent studies using Next-Generation Sequencing (NGS) have suggested that they are not (Mao et al., 2018). Research has increasingly shown that microorganisms may heighten susceptibility to lung cancer (Erb-Downward et al., 2011). Furthermore, antibiotic use has been linked to an increased risk of lung carcinoma, hinting at the oncogenic potential of ecological imbalances (Kim et al., 2023). Microorganisms may also promote lung carcinoma development by inducing inflammation, such as through the activation of Toll-like receptors in immune and epithelial cells (Chen et al., 2022; Orlacchio and Mazzone, 2021). The microbial community in the lung mucosa of the lower respiratory tract shows systematic diversity from that of the upper airways, primarily consisting of genera like *Megasphaera*, *Streptococcus*, *Pseudomonas*, *Fusobacterium*, and *Sphingomonas* (Hilty et al., 2010; Beck et al., 2012).

Previous studies have highlighted the association between lung microbiota and lung carcinoma, yet the specific mechanisms remain poorly understood. In 2016, Lee et al. observed distinct bacterial communities in lung carcinoma compared to benign mass-like lesions (Lee et al., 2016). In 2018, Liu et al. reported a higher abundance of *Streptococcus* in bronchoscopic specimens from lung cancer patients than in controls (Liu et al., 2018). These findings suggest that certain microbiota could help diagnose lung carcinoma types. For instance, Yan et al. discovered significant associations between the genera *Capnocytophaga*, *Selenomonas*, *Veillonella*, and *Neisseria* in saliva samples from lung carcinoma patients and both adenocarcinoma and small cell carcinoma (SCC), indicating their potential as biomarkers (Yan et al., 2015). Interestingly, variations in microbiome composition have been noted among different lung cancer types; for example, squamous cell carcinoma patients with TP53 mutations showed a higher abundance of *Acidovorax*, and the genera *Klebsiella*, *Comamonas*, *Acidovorax*, *Polaromonas*, and *Rhodoferax* were more common in small cell carcinoma than in adenocarcinoma (Yu et al., 2016; Dickson et al., 2014; Greathouse et al., 2018). Although the research is still emerging, some studies suggest that gender may influence the composition of lung microbiota. For example, variations in microbiota have been reported between male and female lung cancer patients, potentially impacting how the disease progresses and responds to treatment. However, the data on gender-specific differences are not yet robust and more research is needed to draw definitive conclusions (Peters et al., 2022).

Bacteria can trigger chronic inflammation by activating pro-inflammatory factors, which in turn promote the proliferation of airway epithelial cells and potentially induce tumor formation (Mao et al., 2018; Laroumagne et al., 2013). Additionally, local microbiota may drive carcinogenesis by directly affecting epithelial cells. In 2019, Jin et al. suggested that local microbiota induce inflammation related to lung carcinoma through the activation of lung-resident γδ T cells. Elimination of symbiotic bacteria reduced lung adenocarcinoma development, as these bacteria promoted the proliferation and activation of Vγ6Vδ1 + γδ T cells, fostering inflammation through the production of effector molecules like IL-17, which in turn stimulates tumor cell proliferation (Jin et al., 2019).

Further research is needed to explore the pulmonary microbiota as a biomarker for the diagnosis and treatment of lung carcinoma. Although links between the pulmonary microbiota and lung cancer have been established, the detailed mechanisms of interaction remain unclear. Unraveling these mechanisms will provide deeper insights into the pathogenesis of lung carcinoma. Moreover, research should aim to accurately control system biases from sampling types and environmental pollutants. Since different sample types, such as lung tissues, sputum, BAL fluid, and bronchoscopy specimens, might contain varying microbial types that influence patient outcomes, establishing unified guidelines for sample collection methods is crucial.

#### Gut microbiota

The gut microbiota comprises diverse bacteria in the human gastrointestinal tract and plays a crucial role in physiology and host health (Dickson et al., 2014; Schuijt et al., 2016). Interestingly, research has established a link between gut microbiota and lung cancer (Routy et al., 2018). Zhuang et al. observed decreased gut microbial function in lung carcinoma cases and identified *Enterococcus* and *Bifidobacterium* as potential biomarkers for the disease (Zhuang et al., 2019). Similarly, Liu et al. found reduced abundance and biodiversity in the gut microbial community among 30 lung carcinoma patients, as well as a decline in probiotic genera (Liu et al., 2019). These differences suggest that gut microbiota may affect the treatment outcomes and prognosis of lung carcinoma.

The gut microbiome composition of lung cancer patients who responded to immune checkpoint inhibitors differed markedly from those who did not respond. Patients with a higher abundance of *Akkermansia muciniphila* showed better responses to anti-PD-L1 therapy; conversely, an abnormal gut microbiota composition was associated with resistance to ICI therapy (Routy et al., 2018). A 2019 study by Jin et al. revealed that in Chinese lung cancer patients responding to nivolumab, gut microbiota was stable and exhibited greater diversity, which correlated with prolonged progression-free survival (PFS) (Jin et al., 2019). A retrospective study involving 118 early-stage lung cancer patients who received immunotherapy indicated that treatment with *Clostridium butyricum* therapy (CBT) significantly extended PFS and overall survival, irrespective of immunotherapy use (Tomita et al., 2020). Additionally, high yogurt consumption was linked to a reduced risk of lung cancer, suggesting protective effects of prebiotics and probiotics against lung carcinoma (Yang et al., 2020).

Moreover, intestinal and pulmonary microorganisms show similar colonization patterns early in life and possess a robust mucosal defense system. The intestinal and lung mucosa share several characteristics, including the ability of goblet cells in both tissues to secrete IgA. Research has indicated mutual influence on immunity between the lungs and intestines (Gill et al., 2010). Studies have shown that short-chain fatty acids (SCFAs), the primary metabolites of gut microbiota from dietary fiber, play roles in regulating both gut microbiota immune functions and lung immunity in allergic models (Cait et al., 2018). Furthermore, it is suggested that lung flora can affect intestinal flora through the bloodstream, linking intestinal and pulmonary microbiota via a complex bidirectional axis involving the lymphatic and blood circulation systems (Renz et al., 2011; Marsland et al., 2015). Disruption of this gut-lung axis can lead to adverse outcomes, including cancer development, pathogen colonization, tissue damage, and an elevated infection risk (Hooper et al., 2012; Mazmanian et al., 2005). This crosstalk offers potential new therapies for lung carcinoma, but further research is needed to explore the mechanisms underlying the gut-lung axis’s influence on lung cancer.

### Advantages and shortcomings

This study provides a focused bibliometric analysis specifically on the microbiota in lung cancer. The primary strength of this research lies in its novelty and the comprehensive methodology employed to map the existing landscape of microbiota research within the context of lung cancer. By concentrating exclusively on this intersection, we have uncovered unique insights and patterns that may not have been apparent in broader reviews of microbiota or cancer independently. This targeted analysis contributes significantly to the understanding of the role of microbiota in oncology, a field increasingly recognized as crucial by the scientific community. The societal impact of this research extends beyond the academic sphere. By identifying key associations between the microbiota and lung cancer, our findings have the potential to inform the development of new clinical applications, including diagnostics and treatments, which could significantly improve patient outcomes. Additionally, the study informs public health policies, enhances scientific education, and raises public awareness of the microbiota’s role in cancer. By highlighting international research collaborations, this work also promotes global scientific cooperation, accelerating progress in understanding and managing lung cancer.

However, our study has notable limitations that must be acknowledged. First, our research scope was restricted to articles indexed in the WoSCC. While WoSCC is a robust and widely used database for scientific publications, this limitation may have led to the exclusion of potentially impactful studies published in other databases or journals not indexed by WoSCC, such as those more regionally focused or newer journals. Secondly, by restricting our analysis to papers written in English, we have inadvertently overlooked significant research contributions documented in other languages. This exclusion might skew the understanding of the field’s true global landscape, as non-English publications could hold critical regional insights or innovative approaches not yet widely discussed in English-language literature. Finally, the methodology employed in this study does not allow for the investigation of species variation among microbes across different countries. Such biological variations require more detailed metagenomic or microbial community analyses, which fall outside the scope of bibliometric analysis. Future studies combining bibliometric trends with biological data could provide a more nuanced understanding of how microbial species related to lung cancer differ geographically.

### Future directions

The limitations outlined suggest a need for a more inclusive approach in future bibliometric analyses. Expanding the search to include multiple databases and considering articles published in multiple languages would enhance the comprehensiveness and accuracy of the analysis. Addressing these gaps could potentially reveal new dimensions and connections in the microbiota-lung cancer nexus that were not captured in this initial study. Moreover, such an expanded approach would better reflect the global effort in this research area, providing a more holistic view of the international scientific community’s contributions.

While our study provides a comprehensive overview of microbiota research in lung cancer, it is important to note that direct studies comparing gut microbiota profiles with survival rates in lung cancer patients are still limited. This gap highlights the need for future research to explore how specific microbial compositions may correlate with long-term treatment outcomes, potentially leading to microbiota-targeted therapies to improve patient survival.

Collectively, while this bibliometric analysis has laid a solid foundation for understanding the role of microbiota in lung cancer, the study’s limitations underscore the importance of broader and more inclusive research strategies moving forward. By adopting such strategies, the scientific community can ensure a more complete and nuanced understanding of how microbiota influences lung cancer, potentially leading to breakthroughs in treatment and diagnosis.

## Conclusion

The microbiota holds significant value and potential for lung cancer research, with publications increasing rapidly each year. Major studies in this field are predominantly conducted in China, the United States, Ireland, and Italy, with journals such as *Cancers* and *Science* playing pivotal roles. Current research focuses primarily on understanding the mechanisms by which lung and/or gut microbiota influence lung cancer, and on developing immune checkpoint inhibitors as therapeutic strategies. Additionally, manipulating the microbiota offers promising new approaches to treating lung carcinoma. The interaction between the gut-lung microbiota axis provides novel therapeutic targets for managing lung carcinoma. However, more research is needed to fully understand the underlying mechanisms connecting the gut-lung axis to lung cancer.

## Data Availability

The original contributions presented in the study are included in the article/Supplementary material, further inquiries can be directed to the corresponding author.
