# Supplementary material for: Global research trends on the associations between the microbiota and lung cancer: a visualization bibliometric analysis (2008–2023)
Source: Front Microbiol. 2024 Aug 30;15:1416385. doi: 10.3389/fmicb.2024.1416385 (PMC11392740; doi:10.3389/fmicb.2024.1416385)
Supplement: Supplementary file 1 [file Data_Sheet_1.PDF]

Supplementary Materials to:

Global Research Trends on the Associations Between the Microbiome and Lung Cancer: A Visualization Analysis (2008-2023)

Maoyuan Zhao[1], Jie Tian[2,3], Wang Hou[4], Liyuan Yin[1], Weimin Li[4,5,6*]

[1] Lung cancer center, Frontiers Science Center for Disease-related Molecular Network, West China Hospital, Sichuan University, Chengdu, Sichuan, China.

[2] Department of Thoracic Surgery, West China Hospital, Sichuan University, No. 37, Guoxue Lane, Wuhou District, Chengdu, Sichuan Province, 610041, China.

[3] Lung Cancer Center, West China Hospital, Sichuan University, Chengdu, Sichuan, China.

[4] Department of Respiratory and Critical Care Medicine, Frontiers Science Center for Disease-related Molecular Network, West China Hospital, Sichuan University, Chengdu, Sichuan, China.

[5] Precision Medicine Research Center, West China Hospital, Sichuan University, Chengdu, Sichuan, China.

[6] Research Units of West China, Chinese Academy of Medical Sciences, West China Hospital, Chengdu, Sichuan, China.

Email: maoyuanzhao2013@outlook.com

Contents

Supplementary Table 1: Top 10 co-cited references on research of microbiota in lung cancer.
Supplementary Table 2: The main research contents of the 20 references with strong citations bursts.

Supplementary Table 1: Top 10 co-cited references on research of microbiota in lung cancer.

Rank	Co-cited reference	Citations	Title	Pubmed ID	Doi
1	Routy B, 2018, science, v359, p91	215	Gut microbiome influences efficacy of PD-1-based immunotherapy against epithelial tumors	29097494	doi: 10.1126/science.aan3706
2	Sivan A, 2015, science, v350, p1084	167	Commensal Bifidobacterium promotes antitumor immunity and facilitates anti-PD-L1 efficacy	26541606	doi: 10.1126/science.aac4255
3	Gopalakrishnan V, 2018, science, v359, p97	161	Gut microbiome modulates response to anti-PD-1 immunotherapy in melanoma patients	29097493	doi: 10.1126/science.aan4236
4	Vétizou M, 2015, science, v350, p1079	138	Anticancer immunotherapy by CTLA-4 blockade relies on the gut microbiota	26541610	doi: 10.1126/science.aad1329
5	Matson V, 2018, science, v359, p104	124	The commensal microbiome is associated with anti-PD-1 efficacy in metastatic melanoma patients	29302014	doi: 10.1126/science.aao3290
6	Iida N, 2013, science, v342, p967	95	Commensal bacteria control cancer response to therapy by modulating the tumor microenvironment	24264989	doi: 10.1126/science.1240527
7	Lee SH, 2016, lung cancer, v102, p89	90	Characterization of microbiome in bronchoalveolar lavage fluid of patients with lung cancer comparing with benign mass like lesions	27987594	doi: 10.1016/j.lungcan.2016.10.016
8	Viaud S, 2013, science, v342, p971	89	The intestinal microbiota modulates the anticancer immune effects of cyclophosphamide	24264990	doi: 10.1126/science.1240537
9	Jin CC, 2019, cell, v176, p998	87	Commensal Microbiota Promote Lung Cancer Development via γδ T Cells	30712876	doi: 10.1016/j.cell.2018.12.040
10	Yu GQ, 2016, genome biol, v17	84	Characterizing human lung tissue microbiota and its relationship to epidemiological and clinical features	27468850	doi: 10.1186/s13059-016-1021-1

Supplementary Table 2: The main research contents of the 20 references with strong citations bursts

Rank	Strength	Main research content	Pubmed ID
1	5.16	Genomic analysis identifies association of Fusobacterium with colorectal carcinoma and reveals alterations in the colorectal cancer microbiota	22009990
2	12.8	Commensal bacteria control cancer response to therapy by modulating the tumor microenvironment	24264989
3	24.37	Commensal Bifidobacterium promotes antitumor immunity and facilitates anti-PD-L1 efficacy	26541606
4	21.66	Anticancer immunotherapy by CTLA-4 blockade relies on the gut microbiota	26541610
5	10.57	The intestinal microbiota modulates the anticancer immune effects of cyclophosphamide	24264990
6	7.85	The potential role of lung microbiota in lung cancer attributed to household coal burning exposures; never smoking lung cancer cases have differing sputum microbiota than controls; bacteria found in sputum may be influenced by environmental exposures associated with the type of coal burned in the home	24895247
7	7.59	The commensal microbiota contributes to the anti-lung cancer response and probiotics co-treatment can enhance the antigrowth and proapoptotic effects of cisplatin	26125762
8	6.45	Fusobacterium nucleatum promotes colorectal carcinogenesis by modulating E-cadherin/β-catenin signaling via its FadA adhesin	23954158
9	6.45	The links between the bacterial microbiota and cancer, with a particular focus on immune responses, dysbiosis, genotoxicity, metabolism and strategies to target the microbiome for cancer prevention.	24132111
10	5.39	Cancer immunology. Mutational landscape determines sensitivity to PD-1 blockade in non-small cell lung cancer	25765070
11	11.11	Discovery and validation of potential bacterial biomarkers for lung cancer	26693063
12	9.36	Among patients with advanced nonsquamous NSCLC that had progressed during or after platinum-based chemotherapy, overall survival was longer with nivolumab than with docetaxel	26412456
13	5.88	Among patients with advanced, previously treated squamous-cell NSCLC, overall survival, response rate, and progression-free survival were significantly better with nivolumab than with docetaxel, regardless of PD-L1 expression level	26028407
14	5.53	Recurrent exposure to certain antibiotics may be associated with cancer risk in specific organ sites.	26338196
15	5.23	Enterococcus hirae and Barnesiella intestinihominis Facilitate Cyclophosphamide-Induced Therapeutic Immunomodulatory Effects	27717798
16	4.82	Fusobacterium nucleatum potentiates intestinal tumorigenesis and modulates the tumor-immune microenvironment	23954159
17	9.96	The differences exist in the bacterial communities of patients with lung cancer and those with benign mass-like lesions. The genera Veillonella and Megasphaera showed the potential to serve as biomarkers to predict lung cancer. Thus, the lung microbiota may change the environment in patients with lung cancer.	27987594
18	9.77	Characterizing human lung tissue microbiota and its relationship to epidemiological and clinical features	27468850
19	5.79	Microbiota modulate tumoral immune surveillance in lung through a γδT17 immune cell-dependent mechanism	24947042
20	4.75	Anticancer effects of the microbiome and its products	28529325
